# Ultrasound- and fluoroscopic-guided, percutaneous cholecystostomy drain placement in canine cadavers, a feasibility and safety study

**DOI:** 10.3389/fvets.2025.1549221

**Published:** 2025-05-09

**Authors:** Jasmin Ordobazari, Charlotte Pfeiffer, Adriano Wang-Leandro, Holger A. Volk, Georga T. Karbe

**Affiliations:** Department of Small Animal Medicine and Surgery, University of Veterinary Medicine, Hanover, Germany

**Keywords:** percutaneous cholecystostomy drain, ultrasound-guided, fluoroscopic-guided, dogs, gallbladder, extra-hepatic biliary obstruction, pancreatitis, cholelithiasis

## Abstract

**Objective:**

To evaluate the feasibility and safety of placing cholecystostomy drains percutaneously under ultrasound and fluoroscopy guidance.

**Study design:**

Experimental cadaveric study.

**Animals:**

Ten canine cadavers.

**Methods:**

Placement of two different locking loop drain systems was tested, an 8F pediatric-nephrostomy (Boston Scientific PNPAS) and a 6.5F SUB-nephrostomy (Norfolk Vet Products). The drains were placed into the gallbladders using a Seldinger-technique under ultrasound and fluoroscopic guidance. After placement, CT-scans were performed to assess drain position, leakage and organ injuries. Anatomic examination was performed to identify and grade iatrogenic injury to the abdominal and thoracic organs. Leak pressures were measured using a water manometer. Procedure time, volume injected and pressure measurements before and at the time of leakage were recorded.

**Results:**

Drain placement into the gallbladder was confirmed by ultrasound and fluoroscopy in 5/5 pediatric-nephrostomy and 0/5 SUB-nephrostomy drains. Mean placement time was 10 min (range 7–12 min) for pediatric-nephrostomy drains. CT-scans confirmed drain placement in 4/5 pediatric-nephrostomy drains, one drain had dislodged. Free abdominal contrast was observed in 4/5 dogs with pediatric-nephrostomy. Drains were placed through the 5th to 10th intercostal space. Anatomic examination showed perforation of the pleural cavity (3/10) for drains placed through the 5th, 7th, and 10th intercostal spaces. Drains passed through the liver parenchyma in the same three dogs. The remaining seven dogs had no organ damage. Pressure testing was performed in the pediatric-nephrostomy drains (4/5). Leakage occurred at a pressure of 4, 9, 12 and 18 cm H_2_O. Leaks were seen at other sites of the gallbladder prior to leaking at the drain entrance point.

**Conclusion:**

Percutaneous cholecystostomy drain placement is feasible in dogs depending on the drain and technique. Risk of pleural space injury must be considered when performing this method. Further studies are needed to establish a safe, standardized percutaneous cholecystostomy technique.

**Clinical significance:**

Imaging-guided, percutaneous cholecystostomy drain placement with the tested method is feasible depending on the drain type. Safety concerns must be addressed prior to clinical application.

## Introduction

Extrahepatic biliary obstruction (EHBO) is a condition caused by a variety of pathologies that ultimately result in the impaired flow of bile from the liver to the duodenum. In both dogs and humans, the condition is associated with a high rate of patient morbidity and mortality. Biliary mucoceles, cholelithiasis, cholecystitis, pancreatitis and neoplasia are common causes of EHBO in dogs, many of which require urgent surgical intervention (1, 2). Reported mortality rates for dogs undergoing various types of biliary surgery range from 0–75% (3–5) and 27–75% specifically for those with EHBO due to pancreatitis (6–10). Post-operative bile peritonitis, persistent hypotension, septic peritonitis, cardiopulmonary arrest, pancreatitis and perceived poor prognosis are reported causes of death or euthanasia (7, 11–15). As biliary obstruction progresses in duration and severity, the patient’s condition deteriorates, increasing both anesthetic and surgical risks (12, 16). Planned surgical intervention has been reported to have a substantially lower mortality rate of 2% compared to 20% mortality in dogs having emergency cholecystectomy (13).

Acute biliary pancreatitis, or gallstone induced pancreatitis is a well-known, potentially fatal condition in people (17, 18). Endoscopic (19) and percutaneous procedures (20, 21) are widely used in humans for urgent relief of biliary obstruction and have considerably decreased patient morbidity and mortality (22–24). Percutaneous cholecystostomy drain placement is an intervention used to stabilize people with acute biliary pancreatitis or gallstones prior to definitive surgery. For this procedure the gallbladder is accessed by passing a needle under imaging guidance into the gallbladder via which a tube is then placed for external bile drainage (25). Indications include the management of cholecystitis, for decompression and diversion of bile and it provides an access portal for the dissolution or removal of gallstones (26).

Early biliary decompression is currently not routinely performed in dogs presenting with EHBO. Endoscopic procedures, although reported (27), are limited in veterinary medicine due to patient size and availability of specialized equipment. There is also a paucity of information regarding the feasibility, safety and clinical applicability of imaging-guided, percutaneous cholecystostomy drain placements in dogs. One case report (28) and one cadaveric study (29) have been published on the use of imaging-guided, percutaneous placement of cholecystostomy drains in dogs. In both reports, locking loop drainage catheters were used, the nephrostomy component from an extra-corporeal bypass system (6.5F SUB-nephrostomy, Norfolk Vet Products, SUB) and a peritoneal drainage system (8F or 10F locking pigtail catheter with trocars, Abscession, Angiodynamis, Queensbury). The latter drains were placed under ultrasound guidance in canine cadavers with low to moderate feasibility (29) and the former was placed under ultrasound- and fluoroscopic-assisted in one Rottweiler (28).

To date, there is no established, safe, imaging-guided method to percutaneously place cholecystostomy drains in dogs. It was therefore the purpose of this study to test the feasibility and safety of placing two different drain systems under ultrasound- and fluoroscopic-guidance. Placement of percutaneous cholecystostomy drains should be minimally invasive, safe, easy, and quick. The hypothesis of this study was that both drain systems can be placed easily and safely.

## Methods and materials

Ten canine cadavers, weighing 5-40 kg, were included. Excluded were dogs with previous hepatobiliary disease, perforating abdominal injuries, tumors in the liver, bile ducts or pancreas. Immediately postmortem, dogs were kept in cold storage and within 24 to 36 h frozen at a temperature of −18°C. In preparation for the study, bodies were thawed over 72 to 84 h at an average room temperature of 20 degrees (30).

Two locking loop drain systems were tested, a 6.5F nephrostomy drain on a stylet of an extra-corporeal bypass system (SUB™-nephrostomy, Locking Loop Catheter with Stiffening Cannula, Norfolk Vet Products, Skokie, Illinois, USA) and an 8F pediatric nephrostomy system (pediatric-nephrostomy, Boston Scientific, Marlborough, Massachusetts, USA; PNPAS). Both drains are tapered at the tip. The placement method was the same for both drain groups, each drain was tested on five consecutive dogs starting with the SUB-nephrostomy. Drain placement was performed by the same two investigators (JO, CP) and were supervised by a diplomate in surgery (GK) and radiology (AW).

**Figure 1:**
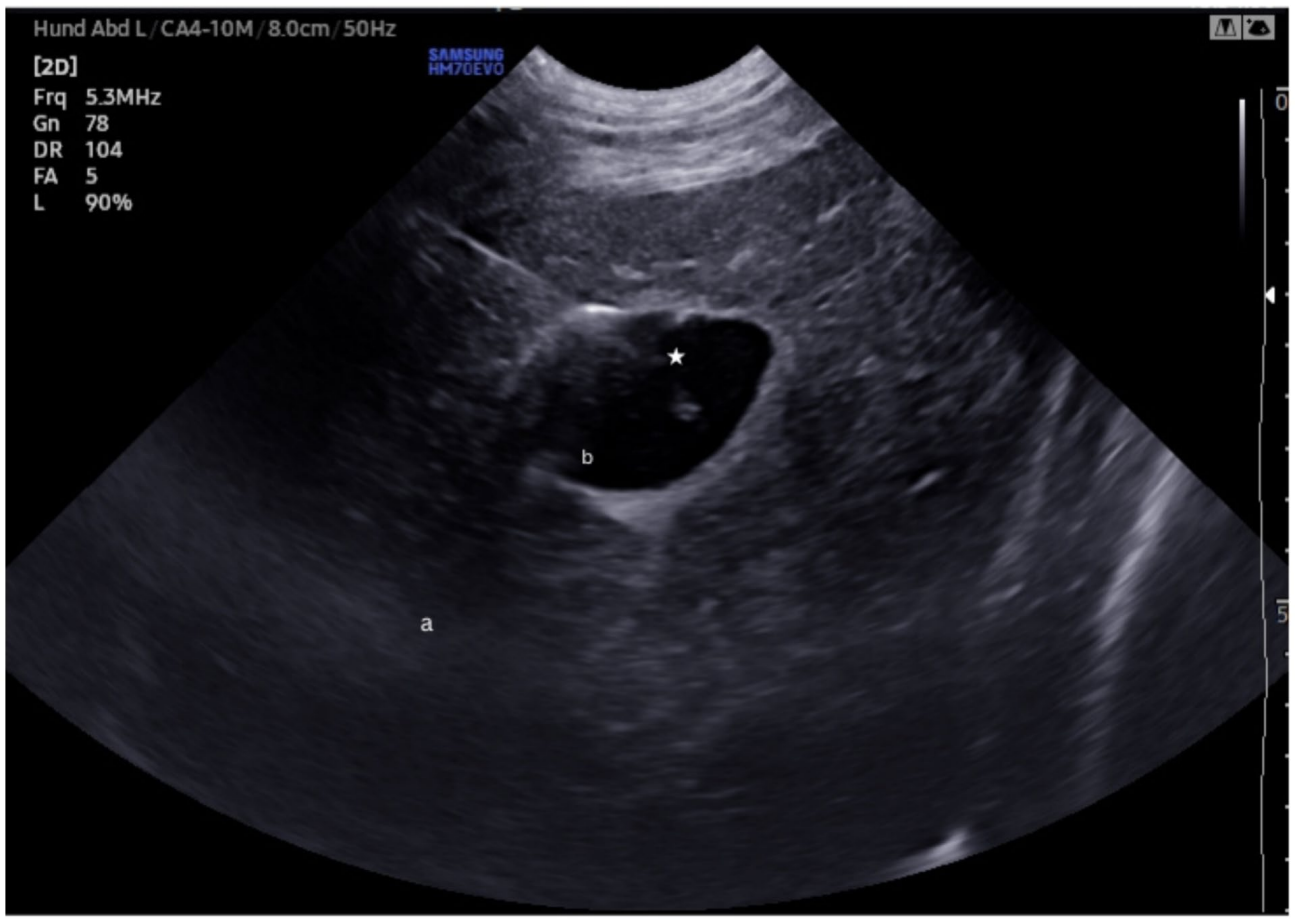
Sonographic picture of the gallbladder- puncture (star). a = liver b = gallbladder.

Dogs were positioned in left lateral recumbency, the fur along the right lateral thorax and abdomen, extending from the 4th rib to the level of the umbilicus, was clipped Overview assessment of the liver and gallbladder to evaluate its location and filling status was performed via subxyphoid acoustic window. The intercostal window was used to assess the best point of cholecystocentesis (Ultrasound device: Samsung HM70 EVO). Holding the transducer in a transverse orientation to the abdomen, the optimal intercostal space for visualizing the gallbladder was identified. At this point, a small skin incision was made, through which either a 20-gauge spinal needle (BD®) or 12-gauge over the needle catheter (MILA- Catheter over needle; 1,211) (Figure 1) was inserted under ultrasound-guidance. For all dogs the same microconvex probe (Samsung CA4-10 M Micro Curved array probe) with a frequency of 5.3 MHz was used, using in-plane technique; depth, gain, and focus were individually adjusted by the operator in each case. Bile was aspirated to confirm needle position followed by injection of water until the gallbladder was moderately distended. Prior to drain placement, 4 mL of 50:50 iobitridol (Xenetix® 350) to water was injected for fluoroscopic visualization. The SUB- and the pediatric-nephrostomy drains were then placed via Seldinger technique under sonographic and fluoroscopic guidance. The total time of procedure from the point of cholecystocentesis to drain fixation was recorded.

### Insertion of the pediatric-nephrostomy

A 0.038″ guidewire was passed through the catheter into the gallbladder to achieve a complete coil within the organ. As provided by the manufacturer, three dilators of increasing size (6F, 8F and 10F) were sequentially passed over the wire through the body wall up to but not through the gallbladder wall, under fluoroscopic guidance. The pediatric-nephrostomy (8F) was then inserted over the guidewire into the gallbladder, the guidewire was removed and the drain secured by its locking loop under fluoroscopic control. Contrast was injected into the gallbladder via the pediatric-nephrostomy to confirm correct placement and assess for leakage. The drain was secured to the abdominal wall with a finger trap suture.

### Insertion of the SUB- nephrostomy

Under fluoroscopic guidance, a 0.035″ J-tip guide wire (Infinity Medical, Nicosia, Cyprus) was passed through the catheter into the gallbladder until the wire coiled once completely within the gallbladder. The spinal needle was removed and a 5F dilator (Mila international, inc.-Medical Instruments for Animals, ZVK-Set) was passed over the guidewire through the body wall up to but not through the gallbladder wall. The dilater was removed and the SUB-nephrostomy on a stylet (6.5F) was passed over the wire into the gallbladder and the locking loop secured under fluoroscopy. Contrast was injected into the gallbladder via the SUB-nephrostomy drain to assess correct placement and leakage. The drain was secured to the body wall with a finger-trap suture.

### Computer tomography

Philips IQon Spectral CT 7500 were performed on dogs where correct drain placement was confirmed on fluoroscopy. The dogs were kept in left lateral recumbency. Immediately prior to scanning 4 mL of 50:50 iobitridol (Xenetix® 350) to water was injected via the drains. Images were assessed by a diplomate in radiology (AW) for drain positioning, iatrogenic organ damage and drain entry points. Free abdominal contrast was scored as either present or absent and subjectively quantified as mild, moderate, or severe.

### Pressure test

**Figure 2:**
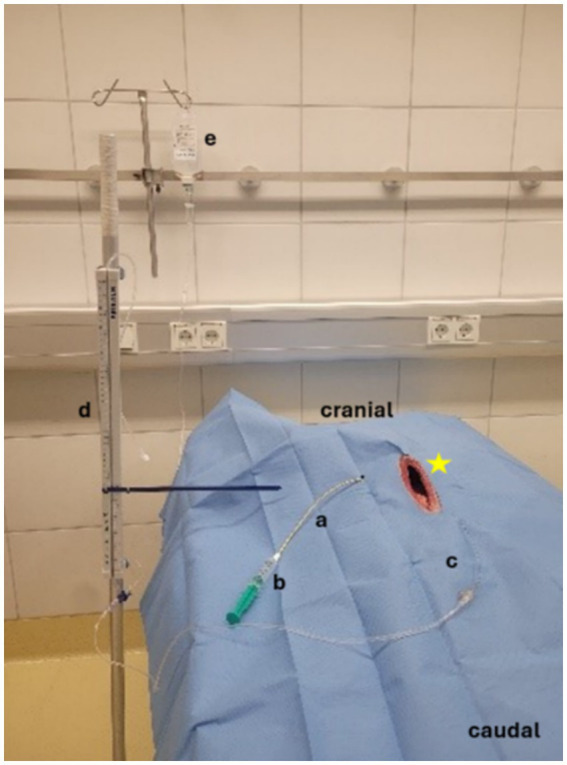
Pressure measurement, Manometer attached at the level of the gallbladder. a = Pediatric-nephrostomy drain, b = 10 mL syringe filled with water, c = feeding tube, d = Water manometer, e = NaCL- Infusion for pressure measurement, star: open abdomen of the dog.

Pressure testing was conducted in dogs with CT confirmation that the drain’s locking loop was fully contained within the gallbladder. A limited ventral midline laparotomy was performed taking care not to interfere with the drain placement. A duodenotomy was performed at the level of the major duodenal papilla through which a 6\u00B0F feeding tube (Fioniavet**®**) was passed into the gallbladder. The cystic duct was then ligated around the catheter with a 3–0 poliglecaprone 25 suture (Ethicon Monocryl, Johnson & Johnson MedTech). A Water- Manometer (Medifix® Measuring bar- B. Braun [4279913], Medifix® Infusion systems [Artikelnummer: 4276116]) was attached to the table at the level of the gallbladder and zeroed. The manometer system was connected to the feeding tube with the tip in the gallbladder. Water was then injected through the percutaneous cholecystostomy drain systems in 10 mL increments. Pressure measurements were recorded and the gallbladder was monitored for leakage. The final pressure and volume before leakage and the pressure and volume at the time leakage were recorded (Figure 2).

### Anatomical dissection

The laparotomy was extended cranially and the diaphragm opened along its ventral attachment for post procedural anatomical examination of the abdomen and thorax. The liver, gallbladder, extrahepatic bile ducts, aorta and caudal vena cava, portal vein the stomach, omentum, pancreas, duodenum and the pleural cavity were inspected for iatrogenic damage. Organ injuries were graded as minor, moderate and major. Minor injuries were defined as minimal injuries, visible only under close inspection that would not require intervention in a clinical patient. Moderate injuries were defined as easily visible, requiring treatment in a clinical patient but not deemed life-threatening. Major injuries were large injuries resulting in a life-threatening condition.

### Statistical analysis

An unpaired t-test was performed to compare the weights between groups. Statistical significance was set at *p* < 0.05.

## Results

Included in the study were four mixed breed and one each of the following breeds: Labrador retriever, Rhodesian ridgeback, Chinese crested, West Highland white terrier, French bulldog and border collie. Dogs in the pediatric-nephrostomy group weighed 5 to15.4 kg and those in the SUB-nephrostomy group weighed 9.8 to 40 kg. An unpaired t-test was performed which showed that the difference in body weights between groups was not statistically significant (*p* = 0.0897).

### Pediatric-nephrostomy

**Figure 3:**
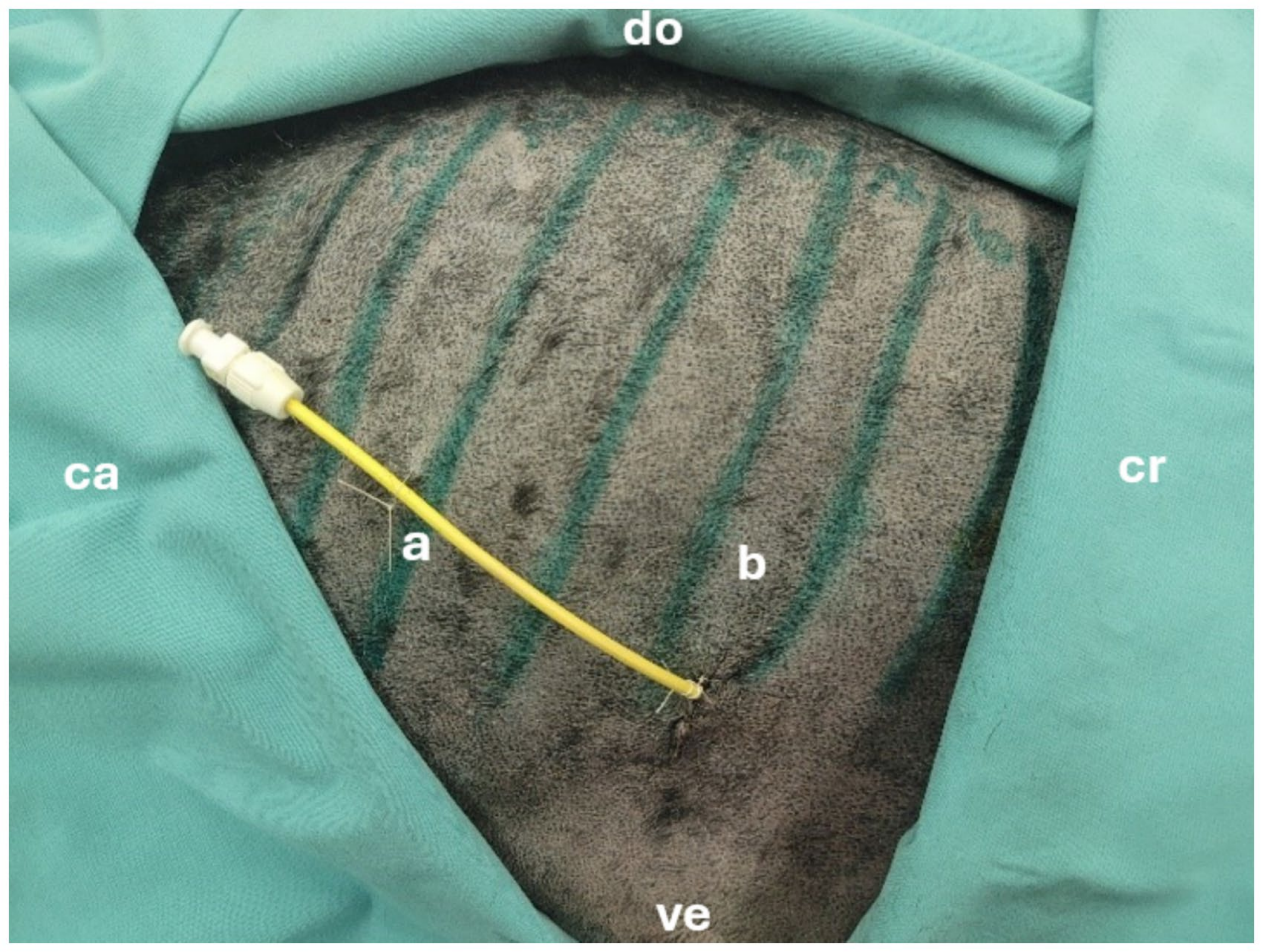
Successfully placed pediatric-nephrostomy in the 8th intercostal space. cr = cranial, ca = caudal, ve = ventral, do = dorsal, a = drain, b = 8th intercostal space.

The pediatric-nephrostomy was placed into the gallbladder in all five dogs (Figure 3) confirmed by fluoroscopy. Percutaneous access points were along the ventral third between the 5th and 10th intercostal space (Table 1). Each gallbladder was punctured only once for cholecystocentesis using a 12-gauge over the needle catheter. Adequate gallbladder distention was achieved by instilling 10 to 25 mL of water prior to drain placement. Procedure times ranged from 7 to 12 min with a median of 10 min.

**Table 1:** Descriptive statistics of all 10 dogs.

Drain type	Breed	Weight (kg)	IC Space	Access point in relation to CCJ	Procedure Time (min)	Leak Pressure (cm H_2_0)	Total Volume at Leak (ml)	Anatomical dissection: Organ injury (grade^*^)	CT Results: Leakage Organ injury
SUB	Chinese crested	5	9th	-		-	-	0	-
SUB	Mixed breed	15.4	7th	-		-	-	Liver (1), Pleura (1)	-
SUB	West highland white terrier	10.2	9th	-		-	-	0	-
SUB	French bulldog	14.6	9th	-		-	-	0	-
SUB	Mixed breed	6.4	7th	-		-	-	0	-
Pediatric	Border collie	19	8th	Above CCJ	10	18	35	0	No leak No injuries
Pediatric	Labrador retriever	18	6th	Above CCJ	8	12	24	0	Mild leak No injuries
Pediatric	Rhodesian ridgeback	40	5th	At level of CCJ	7	-	-	GB (3) Pleura (1), Liver (1)	Moderate leak Pleura Liver
Pediatric	Mixed breed	9,8	10th	Above CCJ	11	9	15	Pleura (1)	Mild leak Pleura
Pediatric	Mixed breed	17,6	8th	Below CCJ	12	4	14	Liver (1)	Mild leak Liver

SUB, SUB-nephrostomy; Pediatric, pediatric-nephrostomy; IC, intercostal space; CCJ, costochondral junction. ^*^Organ damage severity grade: 0: no organ damage, 1: minor, 2: moderate, 3: major. Transhepatic approach with the drain is graded as liver injury grade 1.

**Figure 4:**
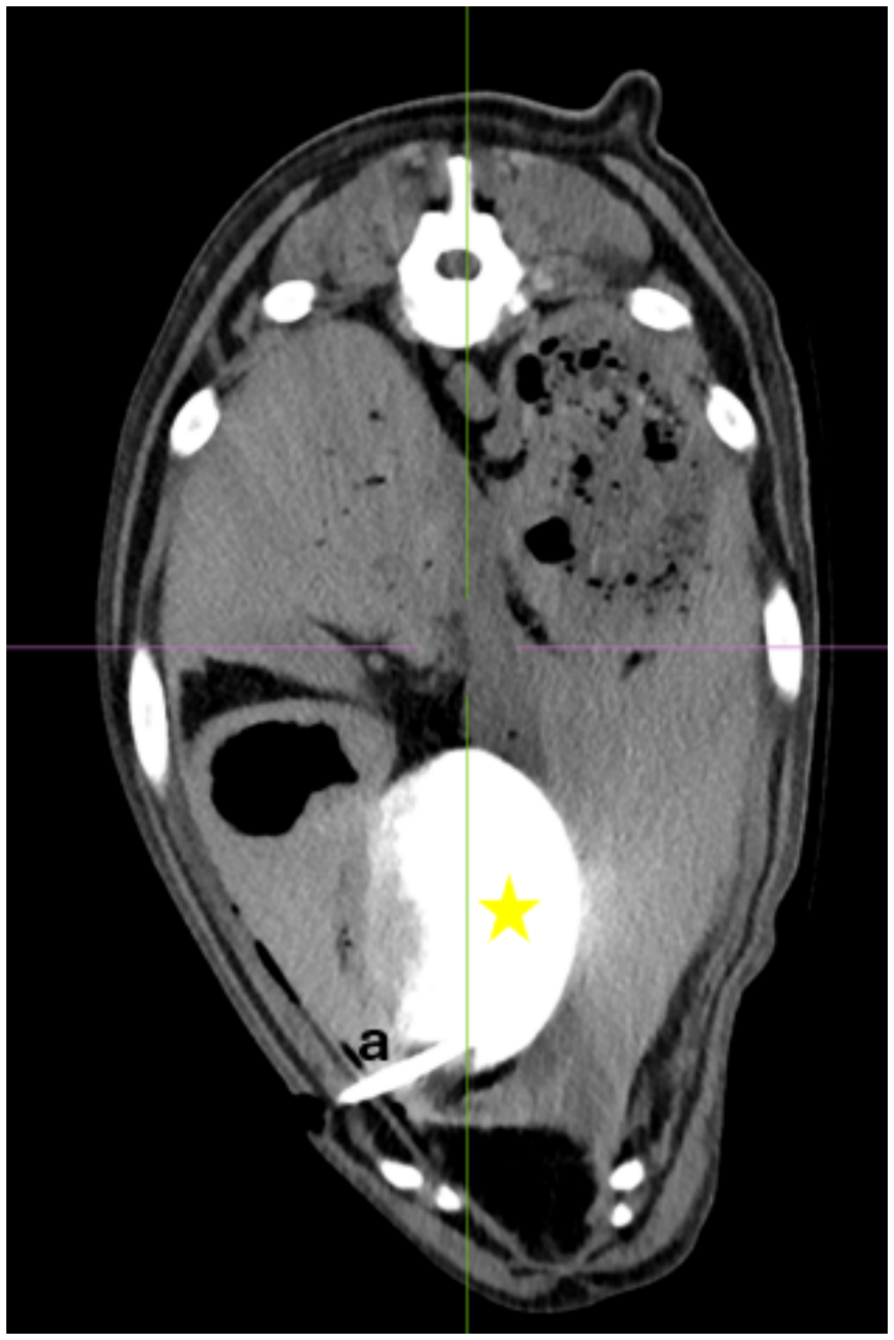
CT-scan image. Star = gallbladder, a = drain entering the gallbladder.

**Figure 5:**
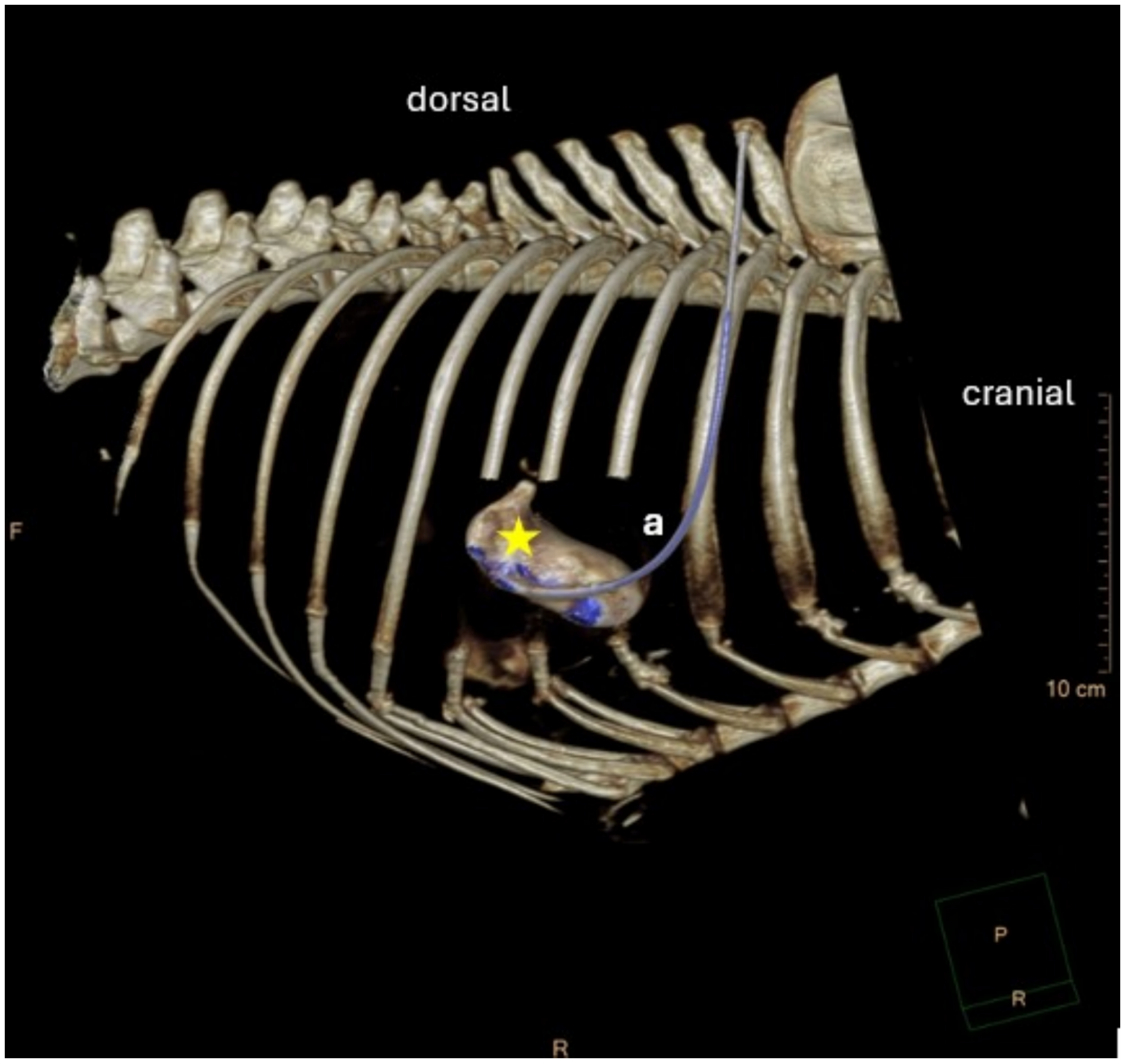
3D-reconstruction of the CT-scan Images. Star = gallbladder, a = drain.

Computer tomography showed four of the five drains to be within the gallbladder (Figures 4, 5). In the fifth dog, the drain was confirmed by fluoroscopy to be within the gallbladder but had dislodged within the two hours until the CT was performed. Only the tip of the drain remained within the gallbladder. Free peritoneal contrast was present in four dogs (Table 1). Pleural space penetration was evident on CT scan in two dogs. In one of these two dogs, the Rhodesian Ridgeback, additional injury to the gallbladder was identified.

**Figure 6:**
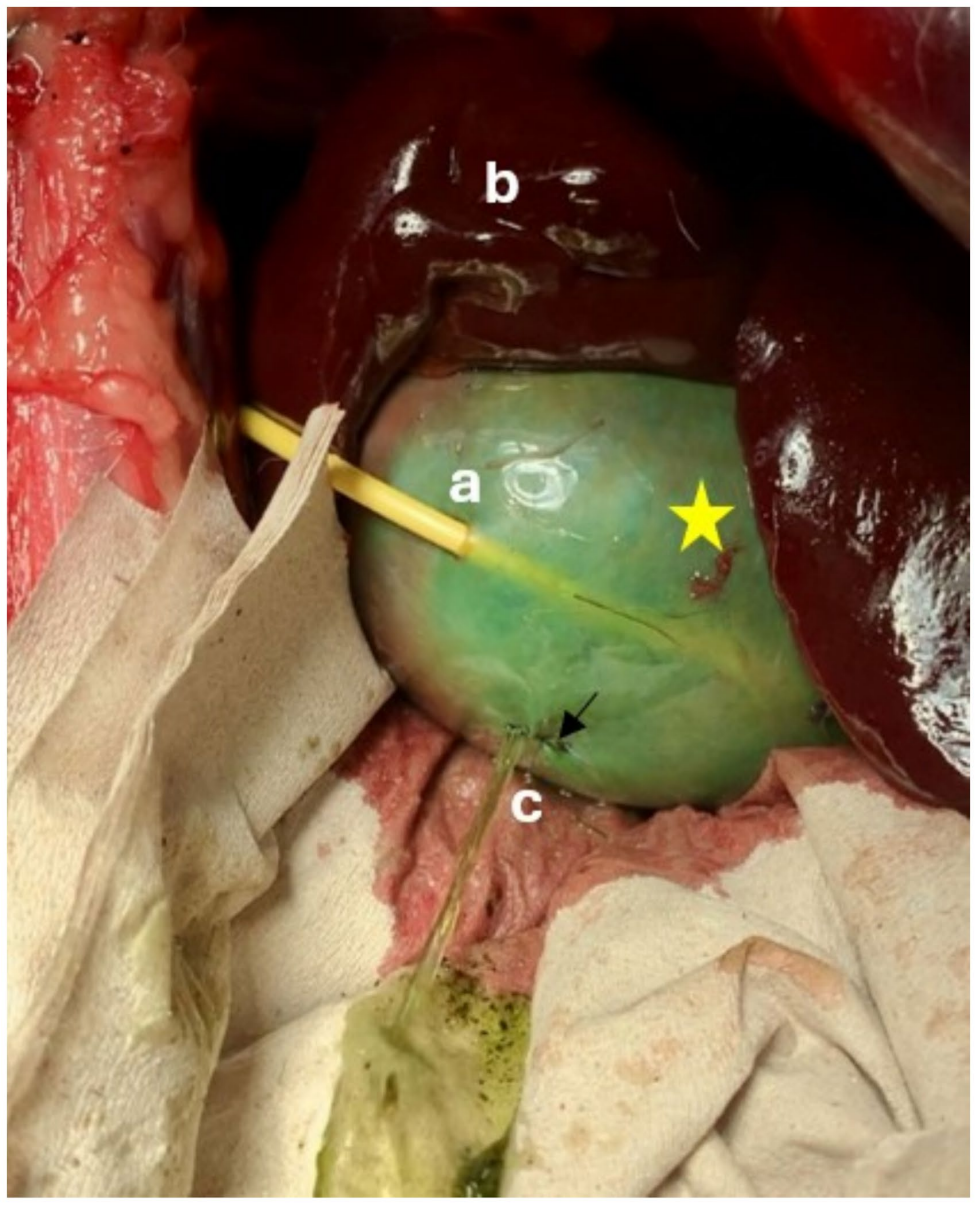
Leak pressure testing of a pediatric-nephrostomy, leakage occurred through a defect in the gallbladder wall that had been oversewn (↓). Defect was presumed to have occurred during passage of the guidewire. Star = Gallbladder, a = Drain entrance site, b = liver, c = Leak site.

Leak pressures ranged from 4 to 18 cm H_2_O. In four dogs, leakage occurred at other sites of the gallbladder prior to leakage at the drain entrance point. These areas were sutured with 4–0 polydiaxanone (Ethicon Johnson& Johnson PDS) (Figure 6) and pressure testing was continued. The gallbladder in one dog was extremely friable and ripped when gently manipulated. Leak pressure testing was therefore not performed in this dog.

**Figure 7:**
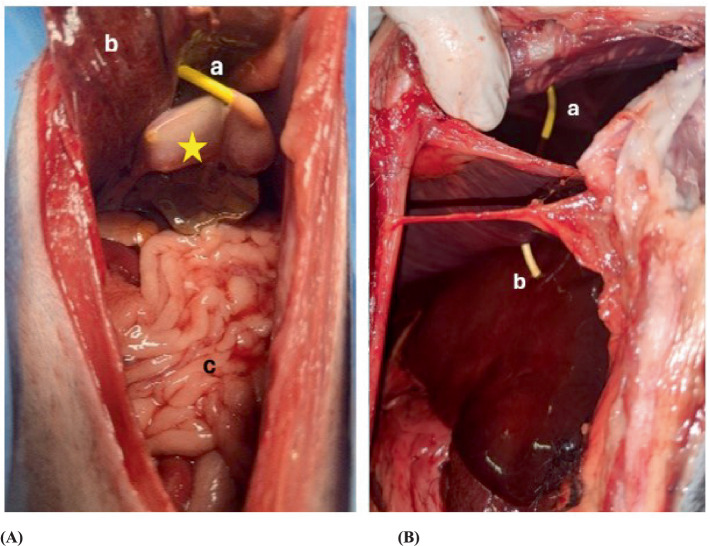
Postmortem examination of a dog where the pediatric-nephrostomy was inserted successfully. **(A)** Shows the drain inserted in the gallbladder in the anatomical dissection. Star = gallbladder, a = pediatric-nephrostomy, b = liver, c = intestine. **(B)** Shows the penetration from the drain of the pleural space and the liver. a = pediatric-nephrostomy through the pleural space, b = pediatric nephrostomy with a transhepatic approach.

The anatomical dissection revealed drainage placement through the right through the right lateral lobe of the liver in two of five dogs (Figure 7A), which were characterized as minor injuries. In the same two dogs, the drainage was through the pleural space. The injury to the pleural space was classified as minor (Figure 7B). Damage to the gallbladder was seen in one dog (Rhodesian Ridgeback), upon gentle retraction of the liver, the gallbladder was found to have a 1 cm defect. During anatomical dissection of the dog with the dislodged pediatric-nephrostomy, the remainder of the drain slipped out of the gallbladder when the body wall was retracted.

### SUB-nephrostomy

The SUB-nephrostomy placement was not feasible in all five dogs. Cholecystocentesis was performed via the 7th intercostal space in two and the 9th intercostal space, along the ventral third. In three dogs a 20-gauge spinal needle was used and a 12-gauge over the needle catheter in two. In the latter two dogs, three attempts were made at cholecystocentesis using the 20-gauge spinal needle, the needle indented the gallbladder wall but did not puncture it. At this point the procedure was modified and a 12-gauge over the needle catheter was used. Each gallbladder was punctured once for cholecystocentesis. The gallbladders were distended with 8 to 30 mL of water. The guidewire was passed into the gallbladder, and the dilator advanced over the wire in all 5 dogs. The SUB-nephrostomy easily passed through the body wall but could not be advanced into the gallbladder. Sonographic control revealed that the drain repeatedly encountered resistance against the gallbladder wall, pushing it away rather than penetrating it. Multiple attempts were made using both slow, controlled and rapid, decisive punctures. Procedure times from the point of cholecystocentesis to advancing the SUB-nephrostomy ranged between 7 and 14 min, further time was not recorded as procedures were eventually aborted. CT scan and leak pressure testing were not performed as drains were not advanced into the gallbladder. Anatomical dissection showed minor damage to the right lateral liver lobe as well as minor damage to the pleura in one dog (Figures 7A,B).

## Discussion

Ultrasound- and fluoroscopically guided percutaneous cholecystostomy drain placement with the described method was feasible with the pediatric-nephrostomy drain system but not with the SUB-nephrostomy. This method resulted in an overall pleural space injury in 30% of dogs (3/10) with more pleural space injury seen in the pediatric-nephrostomy group. These results compare favorably to a previous study, in which only a fifth of percutaneous, ultrasound-guided cholecystostomy drains were placed with 67% resulting in pleural space injury (29).

Pleural space injury occurred in dogs where the gallbladder was access through the 5th, 7th, and 10th intercostal spaces in both drain groups. In contrast, dogs without pleural space injury had drain entry points between the 6th and 9th intercostal spaces. These entry points are more cranial than the 9th to 11th intercostal spaces reported in the previous studies (29), yet resulted in a lower incidence of pleural space injury. Due to the collapsed nature of cadaveric lungs, the prevalence of pulmonary injuries could not be assessed. In a clinical setting, pleural space injury raises safety concerns, including the risk of tension pneumothorax and potential bile contamination of the thoracic cavity. These findings suggest that body conformation, rather than fixed anatomic landmarks, may be a more reliable factor in selecting drain entry points to minimize the risk of thoracic injury when using an intercostal approach. As an alternative to an intercostal approach, a subxyphoid window may eliminate the risk of pleural injury. In all dogs the gallbladders were sonographically examined at this level but cholecystocentesis could not be performed through the subxiphoid window as gallbladders were located further cranially in the ribcage.

Drains traversed liver parenchyma in all three dogs with pleural space injury and in one dog without. Liver injuries were minor and corresponded with a transhepatic placement, aligning with previous reports where pleural space injury occurred in all dogs undergoing intentional transhepatic drain placement (29) through the 9th to 11th intercostal spaces. Transhepatic cholecystocentesis has been suggested as safer with less bile leakage than the direct, transperitoneal or transcholcystic approach, as the liver acts as a natural barrier. A recent meta-analysis comparing transhepatic and transperitoneal percutaneous cholecystostomy in humans found no difference in bile leakage or drain-related complications between the two methods, however risk of bleeding was significantly higher for the transhepatic method (31) he aim of this study was to identify the feasibility of drain placement with this technique rather than evaluate transhepatic drain placement specifically. Leak pressure testing revealed no leakage at the drain entry points, suggesting that transhepatic placement may not be essential. Increased risk of bleeding in patients with coagulation disorders however should be considered when using a transhepatic approach (32), it seems pertinent to consider in patients with impaired coagulation (33, 34).

Bile peritonitis is a key concern with cholecystostomy drains. This study assessed leakage risk through contrast injection during fluoroscopy, CT scan and leak pressure testing. No leakage occurred at the drain-gallbladder interface indicating an effective seal. Small defects, unrelated to the drain entry point may have resulted from needle insertion, guidewire passage, or dilator advancement. While minor bile leakage during drain placement may not pose a critical risk, ongoing leakage can lead to significant morbidity and mortality.

CT scans detected free peritoneal contrast in 80% (4/5) of dogs, though in most cases (3/4) it was subjectively mild. Since additional contrast was introduced into the gallbladders, the observed leakage likely occurred during and after drain placement. Drain dislodgement occurred in one pediatric-nephrostomy drain within two hours of fluoroscopic confirmation. While movement into the CT scanner may have contributed, it was hypothesized that as the gallbladder drained into the duodenum, it gradually decreased in size, causing the gallbladder wall to retracted from the drain. This mode of dislodgement warrants additional investigating as the purpose of cholecystostomy drains is to decompress the gallbladder and biliary tree. Alternatively, drain dislodgement may have resulted from incomplete deployment of the locking loop within the gallbladder during fluoroscopic placement.

The maximum leak pressure in this study (18 cm H_2_O) was lower than previously reported (75 +/− 20 cm H_2_O) (29). However, limited data exists on the pressure threshold needed to prevent cholecystostomy drain leakage. In fasted, healthy dogs, the maximal gallbladder volume is ~1.2 mL/ kg (35), while volumes at leakage in this study ranged from 0.85 to 8 mL/kg. Despite low leak pressures, significant post-drain volume increases are unlikely. The pressure discrepancy is likely due to differences in cadaver storage – this study used frozen cadavers, while the previous study used refrigerated ones. Cold storage affects tissue mechanics, and refrigerated gallbladders may have been more elastic, accommodating higher volumes and pressures.

The drain styles and sizes tested in this study were similar to those previously reported (29). The 8F pediatric-nephrostomy drain is comparable to the trocar tip, locking loop pigtail catheters (8F and 10F) evaluated in a previous cadaveric study (29). In the previous study, only 20% of drains were placed into the gallbladder under ultrasound guidance, whereas the method tested in this study achieved 100% placement using fluoroscopy and 80% confirmed on CT. The improved success rate may be attributed to the use of sequentially increasing dilator sizes, which exceeded the final drain size, compared to the previous study’s use of only a 5F dilator (29). Although the dilators were not passed through the gallbladder wall, they may have created a wider path thereby reducing tissue resistance and facilitating drain advancement into the gallbladder. Dilators were not advanced through the gallbladder wall as there was concern that this would increase risk for bile leakage after drain placement. Difference in needle size used for cholecystocentesis may have also influenced outcomes. The previous study used smaller needles (21 and 22 gauge) (29) whereas larger needles were used in the pediatric nephrostomy drains. Large cholecystocentesis holes may facilitate easier drain passage into the gallbladder.

The use of a smaller dilator may have interfered with the passing of the SUB-nephrostomy, as the 5F dilator is approximately 0.4 mm smaller than the drain itself. It remains unclear however, whether a large dilator would have improved feasibility, given that the SUB-nephrostomy has been successfully placed in a Rottweiler without a dilator (28). In that case, the drain was placed through the liver parenchyma into a dilated hepatic duct and from there advanced into the gallbladder (28). This access route was not possible in the current study, as the cadavers had normal-sized biliary trees. Directly advancing the SUB-nephrostomy through the gallbladder wall was unsuccessful, as the gallbladders repeatedly shifting away from the drain – an issue also observed in the previous cadaveric study (29). The postmortem state of the gallbladders may contribute to this complication. Additionally, in three SUB-nephrostomy dogs, a 20-gauge spinal needle was used for cholecystocentesis instead of a 12-gauge intravenous catheter used for all pediatric-nephrostomy dogs. Puncturing the gallbladder with this smaller needle proved challenging; the flaccid wall of the empty gallbladders indented, requiring significant force to ultimately penetrate it. Consequently, the study design was modified for the remaining cases. Accessing the gallbladder through a large needle hole may have influenced drain passage, potentially explaining the difference in feasibility between drain groups. This represents a limitation of the study.

The cadaveric nature of this study is also a clear limitation, as cadavers without EHBO may not completely mimic a clinical patient. The gallbladder wall was either overly or resistant to puncture in some of the cadavers used. It is likely that post-mortem changes to the gallbladder wall interfered with the placement of the SUB-nephrostomy and with the results from the pressure testing. Although freezing seems to be superior to refrigeration (36) for maintaining soft tissue mechanics, repeating this study in freshly dead cadavers is recommended. Intercostal spaces used for access and pleural space penetration could also have been affected by using cadavers. The lungs were not ventilated which allows the diaphragm to move cranially and with that the liver and gallbladder move further into the ribcage which may be the reason why in the Ridgeback the 5th intercostal space was punctured. In one dog, anatomical dissection revealed a hole in the wall of the gallbladder on the side opposite the drainage site. It is assumed that the gallbladder was very fragile due to its post-mortem condition and therefore the guidewire may have injured the opposite wall when inserted. During pressure measurement, this injury ruptured and a leak developed.

The uneven weight distribution between the two drain groups is a further limitation of this study. Cadavers were collected when available, the first five cadavers were assigned to the SUB-nephrostomy and the next five to the pediatric-nephrostomy group. Dogs in the SUB-nephrostomy group weighed 15 kg or less whereas those in the pediatric-nephrostomy group four of five dogs weighed over 15 kg. An unpaired t-test however showed that the weights were not statistically significantly different between the groups (*p* = 0.897). It could be argued that smaller drains would be better suited in smaller dogs however, the SUB-nephrostomy was previously placed in a 36 kg Rottweiler (28) and a 8F or 10F nephrostomy drain was placed in a Shih Tzu and a domestic shorthair cat (29) suggesting that both implants are suited for large and small dogs. The effect of a learning curve experienced by the investigators could be a further limiting factor to the distribution of successful versus unsuccessful placements between the two drain types.

Small sample size in each group is another limitation of this study. Placement of the pediatric-nephrostomy drain was repeatedly achieved and can therefore be considered as meaningful. Similarly, the SUB-nephrostomy could not be placed in all five dogs with the tested method, further studies are needed to investigate the applicability of this drain system in a clinical setting.

The benefits of percutaneous cholecystostomy drainage are well established in human medicine, where these drains are commonly used pre- and post-operatively to stabilize patients undergoing biliary surgery (24, 37, 38). Similar data is lacking in veterinary medicine, as no safe, minimally invasive, percutaneous method has been established. This study demonstrates that an imaging-guided approach can facilitate drain placement, though safety concerns remain. Careful attention is required to minimize the risk of pleural space injury. Further studies are needed to refine placement methods, identify safe corridors across different canine body conformations and establish standardized protocols for clinical application.

## Data Availability

The original contributions presented in the study are included in the article/supplementary material, further inquiries can be directed to the corresponding author.
